# Endoscopic resection of a granular cell tumor (Abrikossoff’s tumor) in the esophagus using cap-assisted band ligation

**DOI:** 10.1055/a-2088-1849

**Published:** 2023-06-15

**Authors:** Adrian Canavesi, Joaquín Berrueta, Sara Pillajo, Patricia Gaggero, Carolina Olano

**Affiliations:** 1 Department of Gastroenterology, “Prof. Carolina Olano Gossweiler,” Hospital de Clínicas, Universidad de La Republica, Montevideo, Uruguay; 2 Division of Endoscopy, Instituto Nacional de Cancer, Montevideo, Uruguay

Granular cell tumors (GCTs), also known as Abrikossoff’s tumors, are rare neoplasms of Schwann cell origin and are uncommon tumors of the esophagus 1 2 . Depending on their clinical presentation, size, and location, these lesions may need to be resected, either surgically or endoscopically 3 4 5 . A 72-year-old woman with no past medical history of note was found to have a GCT of 10 mm in the esophagus. The patient wished the lesion to be resected.

On endoscopic examination, a yellow submucosal lesion, located at 26 cm from the incisors, was found. The lesion had a squared shape with a flat top, quite typical for a GCT ( Fig. 1 a ). After marking the borders with the tip of a snare using electrocoagulation (60 W), we proceeded to resect the lesion using a cap-assisted device (Duette; Cook, USA) ( Fig. 1 b–d ; Video 1 ).

**Fig. 1:**
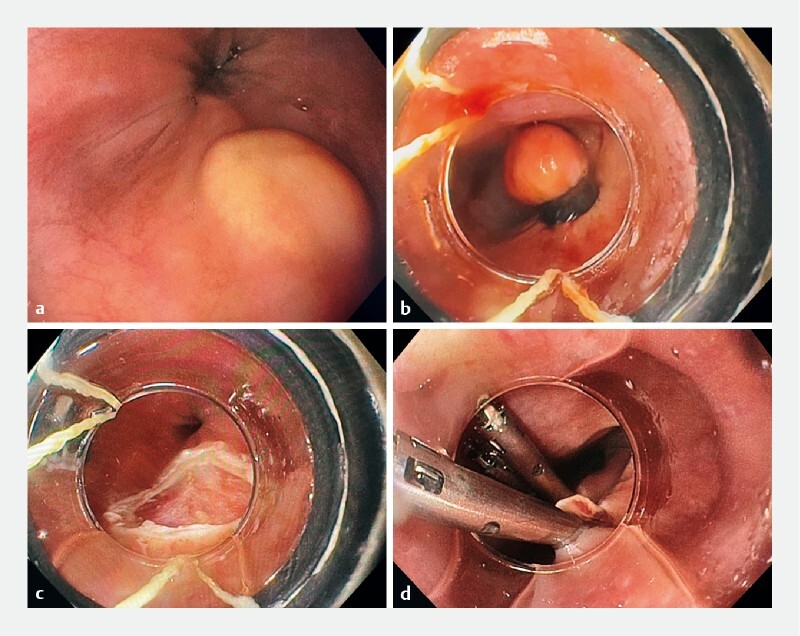
Endoscopic views showing: **a** a granular cell tumor of the esophagus at 26 cm from the incisors; **b** the lesion being aspirated, with two bands released; **c** the tumor resected with a 5-Fr hexagonal snare; **d** closure of the defect with five clips.

**Video 1** Resection of a granular cell tumor of the esophagus using a cap-assisted band device.


The essential aspects of this case were as follows. First, a careful inspection of the lesion, its elasticity, and shape was done. Second, in order to anticipate whether the lesion could be removed, a distal transparent cap was used and the lesion was suctioned inside, revealing that the entire lesion could be aspirated and held inside the cap. Third, markings were applied around the lesion to guarantee subsequent complete lateral resection. Fourth, a cap loaded with bands was used. Histopathology subsequently revealed a well differentiated GCT, S100 positive, with negative margins.

This case stands out for several reasons. First, to the best of our knowledge, this is the first video report of the endoscopic resection of a GCT using a cap-assisted band device. Second, a detailed description of the entire thought and decision-making process for proceeding with band ligation is presented. Generally, these lesions are deep, so more advanced resection methods may be mandatory; however, here we have shown how careful endoscopic interrogation and presuction with a cap allowed us to take the decision to proceed with cap-assisted band ligation.

Endoscopy_UCTN_Code_TTT_1AO_2AG

## Notes

Endoscopy E-Videos

https://eref.thieme.de/e-videos
*E-Videos* is an open access online section of the journal *Endoscopy* , reporting on interesting cases and new techniques in gastroenterological endoscopy. All papers include a high-quality video and are published with a Creative Commons CC-BY license. Endoscopy E-Videos qualify for HINARI discounts and waivers and eligibility is automatically checked during the submission process. We grant 100% waivers to articles whose corresponding authors are based in Group A countries and 50% waivers to those who are based in Group B countries as classified by Research4Life (see: https://www.research4life.org/access/eligibility/ ). This section has its own submission website at https://mc.manuscriptcentral.com/e-videos
